# Tracking progress: an update on animal models for Duchenne muscular dystrophy

**DOI:** 10.1242/dmm.035774

**Published:** 2018-06-13

**Authors:** Dominic J. Wells

**Affiliations:** Department of Comparative Biomedical Sciences, Royal Veterinary College, London NW1 0TU, UK

**Keywords:** Duchenne muscular dystrophy, Dog models, Mouse models, Pig models, Rabbit models

## Abstract

Duchenne muscular dystrophy (DMD) is a progressive, fatal, X-linked monogenic muscle disorder caused by mutations in the *DMD* gene. In order to test treatments for DMD, a range of natural and engineered animal models have been developed, including mice, rats, dogs and pigs. Sui and colleagues have now added a dystrophic rabbit model to this range using CRISPR/Cas9 to disrupt exon 51 of *DMD*. Rabbits have the advantage of being easier to breed and less costly than dog or pig models, but having clear clinical signs, in contrast to many mouse models. There appears to be an effect of body size in models of DMD, as the severity of the clinical signs increases with increasing body size across species. All DMD models have advantages and disadvantages, and it is crucial that investigators understand the limitations of each model when testing novel therapies for DMD in pre-clinical studies.

## Abstract (precis)

**Summary:** With the development of a novel rabbit model of Duchenne muscular dystrophy (dmm032201), this Editorial reflects on the current animal models of this disease, their promise, prospects and pitfalls.

Duchenne muscular dystrophy (DMD) is a progressive, fatal, X-linked monogenic muscle disorder that results in progressive muscle wasting and fibro-fatty replacement of muscle. It affects between 1 in 3500 to 1 in 5000 live male births. The disorder is caused by mutations in *DMD*, a 2.3 Mb gene encoding the 427 kD protein called dystrophin that sits just below the muscle cell membrane (sarcolemma). In DMD, most of the mutations lead to the loss of the open reading frame and thus a failure to produce a stable protein. *DMD* also encodes a variety of smaller isoforms that play important roles in other tissues, including the central nervous system. The full-length dystrophin is believed to have both a structural and signalling role in striated muscle. In the absence of dystrophin, the muscle is prone to damage when contracting, possibly exacerbated by the absence of activity-induced hyperaemia, owing to the mislocalisation of neuronal nitric oxide synthase. The inflammation associated with muscle damage leads to progressive fibrosis, loss of muscle fibres and replacement with fat. DMD is often not diagnosed until 4-5 years of age, although neonatal screening programs can detect massively increased creatine kinase levels leaking from damaged muscles, which is subsequently confirmed by molecular analysis of the *DMD* gene. Prior to diagnosis, affected boys most commonly present with a delay in the major motor milestones and might never run. The weakness of muscles starts proximally, especially in the lower limbs and this leads to the classic Gower's manoeuvre, in which affected boys use their arms to straighten their legs and back when rising from the floor. Untreated patients lose independent ambulation by 13 years of age and many do so much earlier. The heart is also affected, and cardiomyopathy is a problem for all DMD patients. Current medical management includes the use of intermittent or daily steroids, medication to counter the cardiomyopathy, assisted ventilation (especially at night) and spinal surgery, if necessary, to maintain ventilation volume. Corticosteroids slow the rate of disease progression but have significant side effects. Under optimal medical management, patients have an improved quality of life and increased longevity. Currently, patients survive into their late 20s or early 30s on average, but a number of patients survive for more than 40 years. In all cases, patients become progressively weaker and increasingly dependent on their carers.

A number of different approaches are being taken for the development of targeted therapies for DMD, with the aim of preventing disease progression or reversing some of the disease-associated pathology. Although compounds can be screened in cell and tissue cultures, the use of animal models is key to understanding the potential efficacy of different DMD therapies. A wide range of mammalian models have been discovered or generated for DMD. The best known is the dystrophic mdx mouse, which has a premature stop mutation in exon 23 of the murine *Dmd* gene and consequently fails to produce dystrophin (Sicinski et al., 1989). The mdx mouse has been the most widely used model of DMD, with more than 2800 papers published using this mouse. Although the mdx mouse is a good biochemical model of DMD and shows elements of the early stages of the disease, the mice have only a slightly shortened life span and show no obvious clinical signs of muscular dystrophy.

The original mdx has been backcrossed onto a variety of genetic backgrounds that appear very similar to the original mdx mouse. A backcross onto the DBA/2J appears to worsen the phenotype of the dystrophic mouse, such as lower hind limb muscle weight, fewer myofibres, increased fibrosis and fat accumulation, and marked muscle weakness that might be the consequence of reduced regeneration following muscle damage (Fukada et al., 2010; Coley et al., 2016). However, myocardial pathology and hemodynamic defects in the control DBA/2J mice indicate that the DBA/2J-mdx mouse is a poor model of DMD cardiomyopathy (Hakim et al., 2017).

A series of variants with mutations in different parts of the murine *Dmd* were developed (Danko et al., 1992), although some of these retain a low level of full-length dystrophin expression (Danko et al., 1992). Another mouse mutant, lacking many of the smaller isoforms of dystrophin, has been generated by gene targeting: the mdx52 mouse (Araki et al., 1997). Despite some differences from the standard mdx mouse, there is still the problem of limited, if any, clinical signs of muscular dystrophy.

It has been shown that utrophin, an autosomal homologue of dystrophin, precedes the expression of dystrophin during muscle development (Clerk et al., 1993), and it has been suggested that utrophin could substitute, at least in part, for dystrophin. The Davies laboratory has shown that increased utrophin expression can prevent the development of muscular dystrophy in the mdx mouse (Perkins and Davies, 2002). Mdx mice have been crossed with utrophin knockout (KO) mice to produce a double KO, which has a more severe dystrophic phenotype than the mdx mouse (Deconinck et al., 1997). Although this mouse is arguably a better phenocopy of DMD, patients do not actually lack both dystrophin and utrophin. This double-KO model is thus clearly unsuitable for screening all possible therapies for DMD, as those that act via mechanisms that include the upregulation of utrophin will fail to show a therapeutic effect.

Rat models of DMD have been generated recently using genome modification techniques (Larcher et al., 2014; Nakamura et al., 2014). Larcher and colleagues engineered theirs using TALENs targeting exon 23. The muscles showed severe fibrosis and some adipose tissue infiltration, unlike the mdx mouse with the same mutation. The rats showed muscle weakness and decreased activity, as well as evidence of cardiac remodelling and altered diastolic function. Nakamura and colleagues used CRISPR-Cas9 to target exons 3 and 16, producing a range of mutations in the *Dmd* gene in the founder rats. The founder males showed clear evidence of dystrophic pathology and carrier females with mutations in exon 16 successfully produced affected males. Dystrophic rats showed greater fibrosis of the heart at an earlier age than the mdx mouse, but with no clear evidence of functional cardiomyopathy. No subsequent peer-reviewed papers have been published to date for either rat model, although further studies on the Larcher rat have been presented at several meetings.

Cats with dystrophin-deficient muscular dystrophy have been described, but are not suitable as experimental models for human DMD, as they suffer from hypertrophy of the diaphragm and tongue, which makes eating and drinking very difficult for the affected animals (Gaschen et al., 1999).

A number of different breeds of dogs presented at veterinary clinics have been diagnosed with dystrophin-deficient muscular dystrophy. Several of these have been subsequently used to develop dog models of DMD, the most common being a Golden Retriever with Muscular Dystrophy (GRMD), also known as CXMD, which carries a splice site mutation that leads to the loss of exon 7 and thus a failure to produce dystrophin (Sharp et al., 1992). This model has been distributed to a number of colonies worldwide, including sites in the USA, France and Brazil. While these dogs do show clear clinical signs analogous with DMD, there are some significant differences. A number of the GRMD dogs die within the first 6 months of life and there is considerable dog-to-dog variation, which, with the small numbers available for each experimental group, makes it difficult to show clear statistically significant results. In contrast to GRMD dogs, DMD neonates do not show enhanced early mortality.

Another dog model (deltaE50-MD dog), carrying a splice site mutation leading to the loss of exon 50, is under development at the Royal Veterinary College (London, UK) based on an index case identified in 2009 (Walmsley et al., 2010). A carrier half-sister of the index case has been bred onto the Beagle background and the colony is currently undergoing a natural history study. Preliminary data were presented in a series of posters at the 2018 UK Neuromuscular Translational Research Conference (Riddell et al., 2018). To date, the phenotype of the affected deltaE50-MD dogs has proved fairly consistent, with clear clinical signs of the disease and without an increased neonatal mortality.

The pig is an attractive option for translational studies, as it is a very similar size to humans and could thus be a good test of potential problems when scaling up a therapy first tested in mice before translating it for use in humans. Klymiuk et al. (2013) used gene targeting to delete exon 52 of the porcine *DMD* gene. However, these pigs die prematurely, often in the first week of life, and survival appears to depend on the level of utrophin expression. None of the pigs survive to breeding age and, to date, the production of a carrier female has not been described.

Both dogs and pigs have immune systems that are closer to humans, eat a diet more similar to ours and are of comparable sizes, particularly to the pre-teenage years in humans, compared with rodent models. In addition, large animal models of DMD clearly demonstrate clinical signs of disease that can be used to assess response to treatment in tests that have translational value for humans. However, the use of such models is not without problems. Dogs only come into oestrus twice a year and gestation is on average 63 days. Pigs can be bred all year round but the gestation period is even longer, at 114 days. Thus, breeding animals for experiments takes considerably longer than mice (gestation period of 21 days) or rats (gestation period of 21 to 24 days). Not only does it take longer for large animals to reproduce, but they also require considerable space per animal and have additional requirements for social interaction with humans, all of which substantially increases the maintenance costs per animal. Importantly, public perceptions of value differ between the species with a high affinity with dogs, and therefore an increased reluctance to see them being used as experimental animals compared with public attitudes towards rodents.

Intriguingly, Sui et al. (2018) have just published a description of a rabbit model for DMD. They used a CRISPR-Cas9 to target exon 51 of the *Dmd* gene to ablate dystrophin expression in a New Zealand rabbit. About 20% of the DMD KO rabbits died within the first 2 weeks after birth, and approximately half (42.6%) died by 20 weeks of age. The muscles of the surviving DMD KO rabbits show histological pathology typical of DMD, although the degree of fibre loss and fatty replacement was not quantified in this study. The left ventricular ejection fraction and fraction shortening of the DMD KO rabbits were significantly decreased compared with the control rabbits at 16 weeks of age, suggestive of a developing cardiomyopathy. Finally, DMD KO rabbits exhibited significantly reduced mobility and inability to climb a step. Mobility was measured using a fitness tracker and similar analyses of daily activity have been proposed for human clinical trials.

Rabbits breed throughout the year in captivity and gestation is only 31 days, thus large numbers of animals can be bred rapidly. Although the dystrophic rabbit is an improved model compared with the various mouse models, as the rabbits have clear clinical signs of the disease but are substantially easier to breed and are less expensive than dog models, it is not entirely clear how different this DMD rabbit is from the rat models. Importantly, the rabbit model shows clear cardiomyopathy, whereas there was no change in the ejection fraction or fraction shortening in the only dystrophic rat paper that reported cardiac function (Larcher et al., 2014). Unlike the rat models, the rabbit described by Sui and colleagues has a high mortality by 5 months and the reasons for this should be investigated further. If this mortality is caused by cardiomyopathy, as suggested by the authors, then the rabbit model could prove to be the model of choice for testing therapeutic drugs for the dystrophic heart.

So where are we now with animal models of DMD? We now have mouse, rat, rabbit, pig and dog models of DMD, each with their own particular properties. Is one model better than all the others? The answer is clearly no. Rodent models allow us to breed sufficient numbers to power statistically robust studies, but they have limited clinical signs that cannot be directly extrapolated to DMD. General activity appears lower in rodent and the newly developed DMD rabbit model, but it is not clear whether this reduction is sufficiently robust for accurate drug screening, as some candidate drugs might inhibit activity by, for example, inducing nausea; or increase activity by, for example, stimulating the brain. Conversely, dog models, in which we can undertake clinically relevant endpoints, such as the 6-min walk distance and timed function tests, are difficult to breed in sufficient numbers and are exceedingly expensive, which limits the statistical power in the experiments. In some models, such as the dystrophic rabbit, pig and GRMD, there is significant early mortality, which does not resemble the human condition. There appears to be an effect of body size, whereby the severity of clinical signs increases with increasing body size across the species. Indeed, within the dog breeds, the phenotype of the GRMD mutation is less severe when bred onto the smaller Beagle background (Walmsley et al., 2010). Perhaps we need to consider which stages of DMD we intend to model by the different animals. A consideration based not on the relative age of each species, but on their pathological presentation could be more relevant to their translational impact. Hence, the mdx mouse is a model of the neonate to 3-year-old DMD boy, whereas the GRMD dog model is closer to a 5- to 10-year-old DMD boy. The new rabbit model generated by Sui and colleagues might fill a gap between the mouse and the dog, and could prove to be particularly useful for testing drugs that can slow the cardiomyopathy, as this is currently a major cause of death for patients with DMD. It is an exciting time in DMD with a number of novel therapies entering the clinic and even more in the pipeline. Owing to the rarity of DMD, it is essential that we select only the most promising therapies for clinical testing so as not to exhaust our limited number of the most informative patients. A clear understanding of the limitations of each animal model is vital for accurate interpretation of the results of any pre-clinical therapeutic intervention and the potential for translation into the clinic for the treatment of DMD.
